# Supplementary material for: Adipocyte-Mediated Electrophysiological Remodeling of PKP-2 Mutant Human Pluripotent Stem Cell-Derived Cardiomyocytes
Source: Biomedicines. 2024 Nov 14;12(11):2601. doi: 10.3390/biomedicines12112601 (PMC11592320; doi:10.3390/biomedicines12112601)
Supplement: Supplementary file 1 [file biomedicines-12-02601-s001.zip › biomedicines-3277674-supplementary.pdf]

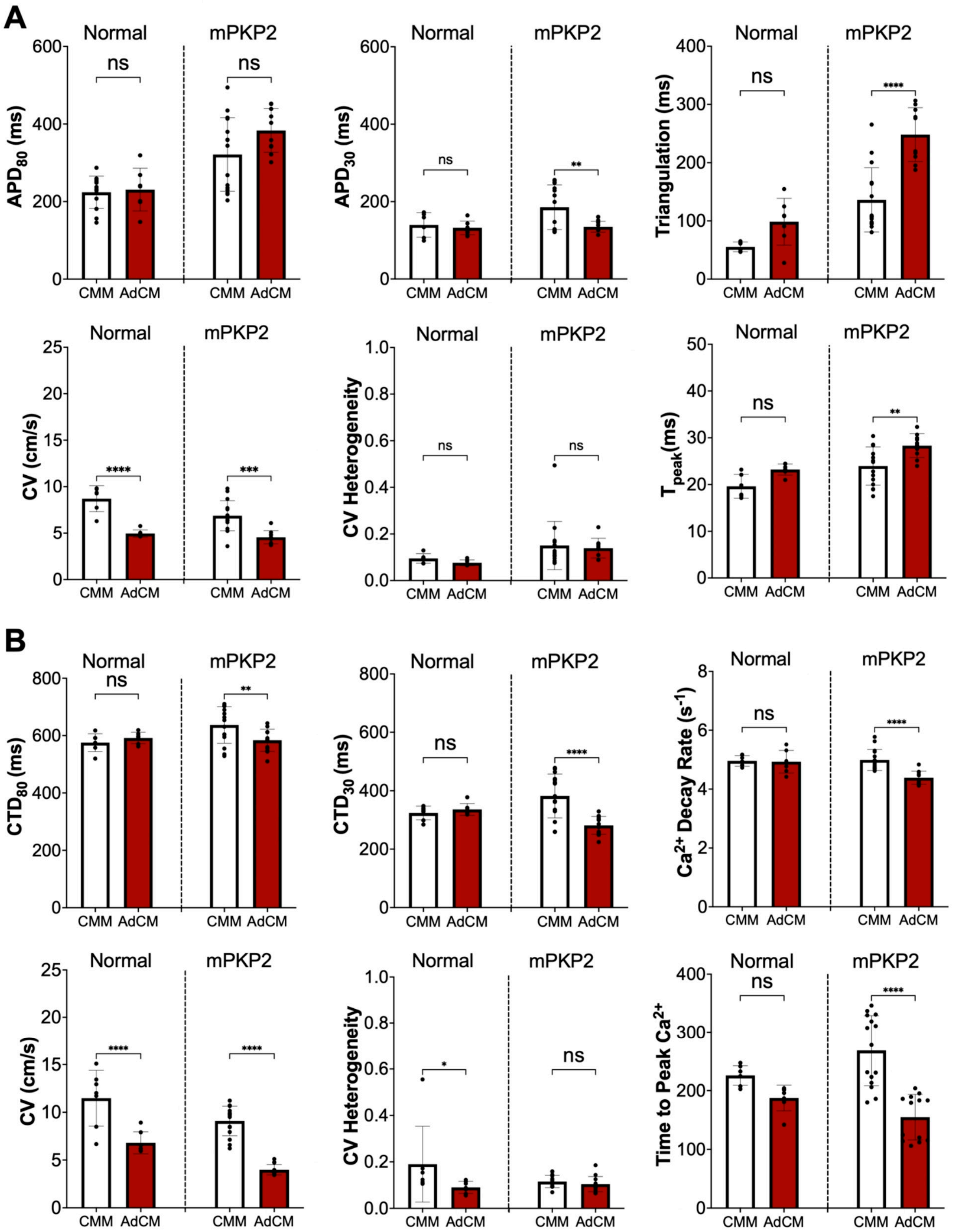

Supplemental Figure S1: Optical mapping of normal and mPKP2 hiPSC-CMs cultured in CMM or in AdCM for 24 hr. (**A**) Graphs summarizing averaged transmembrane voltage mapping data from normal and mPKP2 hiPSC-CMs. Electrophysiological characterizations include action potential duration (APD_{80} and APD_{30}), triangulation, conduction velocity (CV), CV heterogeneity, and maximum T_{peak} (ms). (**B**) Graphs showing calcium transients and include values for calcium transient duration at 80% recovery (CTD_{80}), calcium transient duration at 30% recovery (CTD_{30}), Ca^{2+} decay rate, conduction velocity (CV), CV heterogeneity of all measurements within each sample), and time-to-peak Ca^{2}. Data are expressed as mean ± SD, n = 3, and significance for comparisons between normal and mPKP2 hiPSC-CMs is as follows: * $p < 0.05$ **$p < 0.01$ ***$p < 0.001$ **** $p < 0.0001$.

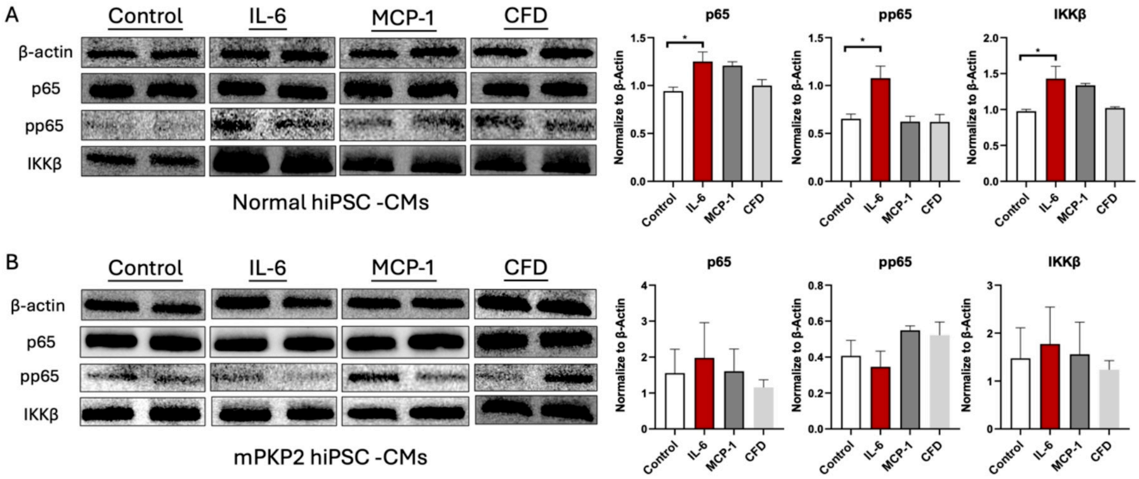

Supplemental Figure S2: Effect of cytokines on NF-kB signaling in normal (JHU001) or mPKP2 (398-100) hiPSC-CMs. (**A**) Representative Western blots for normal hiPSC-CMs and quantified data (bar graphs) following normalization to β-actin. (**B**) Representative Western blots for mPKP2 hiPSC-CMs, and quantified data following normalization to β-actin. Data are expressed as mean ± SD, n = 3, and significance as indicated: *$p < 0.05$. Abbreviations: RelA/p65 nuclear factor, NF-κB; pp65 (phosphorylated p65); IKKβ (IkappaB kinase); CMM (cardiomyocyte maturation medium); AdCM (adipocyte- conditioned medium).
